# Lattice Symmetry and Identification—The Fundamental Role of Reduced Cells in Materials Characterization

**DOI:** 10.6028/jres.106.050

**Published:** 2001-12-01

**Authors:** Alan D. Mighell

**Affiliations:** National Institute of Standards and Technology, Gaithersburg, MD 20899-8520

**Keywords:** crystallography, identification, mathematical lattices, reduction, symmetry determination

## Abstract

In theory, physical crystals can be represented by idealized mathematical lattices. Under appropriate conditions, these representations can be used for a variety of purposes such as identifying, classifying, and understanding the physical properties of materials. Critical to these applications is the ability to construct a *unique* representation of the lattice. The vital link that enabled this theory to be realized in practice was provided by the 1970 paper on the determination of reduced cells. This seminal paper led to a mathematical approach to lattice analysis initially based on systematic reduction procedures and the use of standard cells. Subsequently, the process evolved to a matrix approach based on group theory and linear algebra that offered a more abstract and powerful way to look at lattices and their properties. Application of the reduced cell to both database work and laboratory research at NIST was immediately successful. Currently, this cell and/or procedures based on reduction are widely and routinely used by the general scientific community: (i) for calculating standard cells for the reporting of crystalline materials, (ii) for classifying materials, (iii) in crystallographic database work (iv) in routine x-ray and neutron diffractometry, and (v) in general crystallographic research. Especially important is its use in symmetry determination and in identification. The focus herein is on the role of the reduced cell in lattice symmetry determination.

## 1. Introduction

In theory, physical crystals can be represented by idealized mathematical lattices. Under appropriate conditions, these representations can be used for a variety of purposes such as identifying, classifying, and understanding the physical properties of materials. Critical to these applications is the ability to construct a *unique* [1] representation of the lattice. The vital link that enabled this theory to be realized in practice was provided by a 1970 paper on the determination of reduced cells by Santoro and Mighell [2]. This seminal paper led to a mathematical approach to lattice analysis initially based on a systematic reduction procedure and the use of standard cells. Subsequently, the process evolved to a matrix approach by Karen and Mighell [3,4] based on group theory and linear algebra that offered a more abstract and powerful way look at lattices and their properties.

Conceptually, the reduced cell is a unique primitive cell based on the shortest three lattice translations. As it can be determined from any cell of any lattice and because it has an exact mathematical definition, it can be used as a *standard* cell. As such in one way or another it has been widely accepted and is routinely used in virtually every crystallographic laboratory worldwide. Application of this cell to both our database work and our laboratory research at NIST was immediately successful. Currently, this cell and/or procedures based on reduction are extensively used: (i) in calculating standard cells for the reporting of crystalline materials, (ii) in classifying materials, (iii) in crystallographic database work (iv) in routine x-ray and neutron diffractometry, and (v) in general crystallographic research. Especially important is its use in identification and in symmetry determination.

### 1.1 Identification

At NIST, a new and highly selective analytical method for the identification of crystalline compounds was created [5–7]. In practice, this procedure—based on cell/element type matching of the unknown against a file of known materials represented by their respective reduced cells—has proved an extremely practical and reliable technique to identify unknown materials. The uniqueness of the procedure was first demonstrated using the NBS TODARS System (Terminal Oriented Data and Analysis and Retrieval System) at the Clemson ACA Meeting in 1976.

Today the scientific community routinely uses this identification strategy, as it has been integrated into commercial x-ray diffractometers [8]. In addition, the identification procedure—integrated into database distribution software—is routinely used in identifying unknowns against the various crystallographic databases. Finally, because of its high selectivity, the method plays a critical role in the linking of data on a given material that appears in different scientific databases. This ability paves the way to the efficient use of multiple databases in the knowledge-based design and characterization of new materials.

### 1.2 Symmetry Determination

Because the reduced cell is a *unique* standard cell, it can be used to determine the metric symmetry of a material as described by Mighell, Santoro, and Donnay in the *International Tables for X-Ray Crystallography* [9]. The focus of this paper will be on the role of the reduced cell and form in symmetry determination of an *original* lattice and of the associated derivative lattices. In addition, the impact of specialized reduced forms on lattice properties such as lattice metric singularities will be analyzed. Research has shown that there exists a close link between metric and crystal symmetry. Consequently, symmetry determination procedures based on reduction and reduced forms are widely used in the software that is associated with automated x-ray diffractometers. Likewise they are used by the crystallographic data centers to critically evaluate symmetry.

## 2. Determination of the Bravais Lattice and Conventional Cell

A cell is reduced provided it satisfies both the main and special conditions for reduction as given in Table 1. The main conditions are used to establish that a cell is based on the three shortest lattice translations whereas the special conditions serve to select a unique cell when two or more cells in the lattice have the same numerical values for the cell edges. Procedures and transformation matrices for calculating this cell are given in [2] and in Karen and Mighell [6] and are incorporated into the computer program NIST*LATTICE [10]. From the reduced cell, the reduced form (***a***·***a·b***·***b·c***·***c***/***b***·***c·a***·***c·a***·***b***) is determined which can then be used to determine the metric symmetry^1^ of the lattice via table lookup procedures. As the metric symmetry is highly correlated with the crystal symmetry, such lookup procedures are widely used in modern crystallography—e.g. in automated single-crystal x-ray diffractometers.

### 2.1 Classification of the 44 Reduced Forms

In Table 2, the 44 reduced forms and the corresponding conventional cells are presented in a simple format. This table is a slight modification of Table 5.1.3.1, which was published in the International Tables for X-Ray Crystallography (1969) [9]. It is a shortened version with appropriate errata and addenda [11,12,13]. Table 2 gives the transformation matrices relating the reduced cell to the corresponding conventional cell. For convenience, the reduced forms are grouped into four categories: 1) *a* = *b* = *c*; 2) *a* = *b* ≤ *c*; 3) *a* ≤ *b* = *c*; and 4) *a* ≤ *b* ≤ *c* (i.e., no special conditions other than those required for the reduced cell). Within each category, the reduced forms are further divided into positive and negative reduced forms. To match a reduced form against the table, one starts with the highest possible category and works down. Once a match is found, the experimentalist can transform the reduced cell to the conventional cell using the matrix given in the last column of the table.

### 2.2 Rules for the Conventional Cells

The conventional cells obtained by using Table 2 are logical cells for the reporting of crystallographic results as they are based on both symmetry and metric considerations. For cell edges not defined by symmetry, the shortest edges are used. The following conventions apply: (1) In the hexagonal and tetragonal systems, *c* is taken as the unique axis. (2) In the rhombohedral system, the triply primitive hexagonal cell is used. (3) In the orthorhombic system, the axes of the primitive, body-centered, and face-centered cells are labeled to obey *a* < *b* < *c*. The side-centered cell is taken as C-centered with *a* < *b*. (4) In the monoclinic system, *b* is taken as the *unique* axis, and *a* and *c* are chosen coincident with the shortest two translations in the net perpendicular to *b*. (To assure the shortest translations, the conditions in the footnote for the specified centered monoclinic lattices must be checked. In those cases for which the transformation matrix in the footnote premultiplies a given table matrix, the resultant cell centering is indicated in parentheses following the transformation matrix.) The angle *β* is taken to be non-acute. This choice allows primitive, side-centered, and body-centered cells. In the primitive and body-centered cells *a* and *c* obey *a* < *c*. The side-centered cell is taken as C-centered. (5) In the triclinic system, the conventional cell is the reduced cell with *a* ≤ *b* ≤ *c*.

### 2.3 Adoption of the Conventional Cells by the Scientific Community

Today, conventional cells as specified above for Table 2—or closely related cells—are widely used for the reporting of crystallographic results in the scientific literature. For example, this is true for almost all structures reported in the Journals *ChemCom* and *Acta Crystallographica C*. As soon as it was published, Table 2 was integrated into the software associated with automated single-crystal x-ray diffractometers that collect the data. In addition, to facilitate the use of these conventions, Volume A of the *International Tables for Crystallography* has been expanded to give explicitly the required space group settings in the monoclinic system (e.g. the atomic positions in an I-centered cell).

The widespread acceptance and use of these conventions has had a major scientific impact both in solving structures and interpreting the results of structure determinations. For example, use of the conventions has made structure determination more efficient, prevented duplicate structure determinations, helped to eliminate errors in symmetry determination, and helped prevent confusion especially in working with monoclinic structures, which include approximately 70 % of all organic and organometallic crystalline compounds.

## 3. Population Statistics

The reduced cell and reduced form are routinely calculated using NBS*AIDS83 [14] for all compounds entering the various crystallographic databases including the Cambridge Structural Database [15], and the ICDD Powder Diffraction File [16] and NIST Crystal Data [17]. For organic compounds in NIST Crystal Data, Tables 3, 4, and 5 give the *metric lattice* statistics for the 44 reduced forms, the 14 Bravais lattices, and the 7 crystal systems, respectively. These results are in sharp contrast to the corresponding population statistics for inorganic materials for which the higher symmetry reduced forms, Bravais lattices and crystal systems are more heavily populated.

Table 3 shows that most organic compounds (82 %) crystallize in lattices that are characterized by only 6 of the 44 reduced forms (31–35, 44). Furthermore, collectively many materials (9.5 %) crystallize in the 13 side-centered monoclinic reduced forms (10, 14, 17, 20, 25, 27–30, 37, 39, 41, and 43). Table 4 shows that most organic compounds crystallize in a triclinic, mono-clinic, or orthorhombic Bravais lattice with the primitive lattice (87.1 %) by far the most common. Table 5 shows the distribution by crystal system. Only 5.8 % of organic compounds are in the higher symmetry—rhombohedral, tetragonal, hexagonal and cubic—crystal systems. A comparison of the statistics with those reported earlier [18] shows the same general distribution. However, as molecules studied become larger and more complex, the triclinic system becomes more common (19.8 % vs 15 %).

## 4. Applications in Routine Diffractometry and in Data Evaluation

### 4.1 Routine Diffractometry

**Fig. 1:**
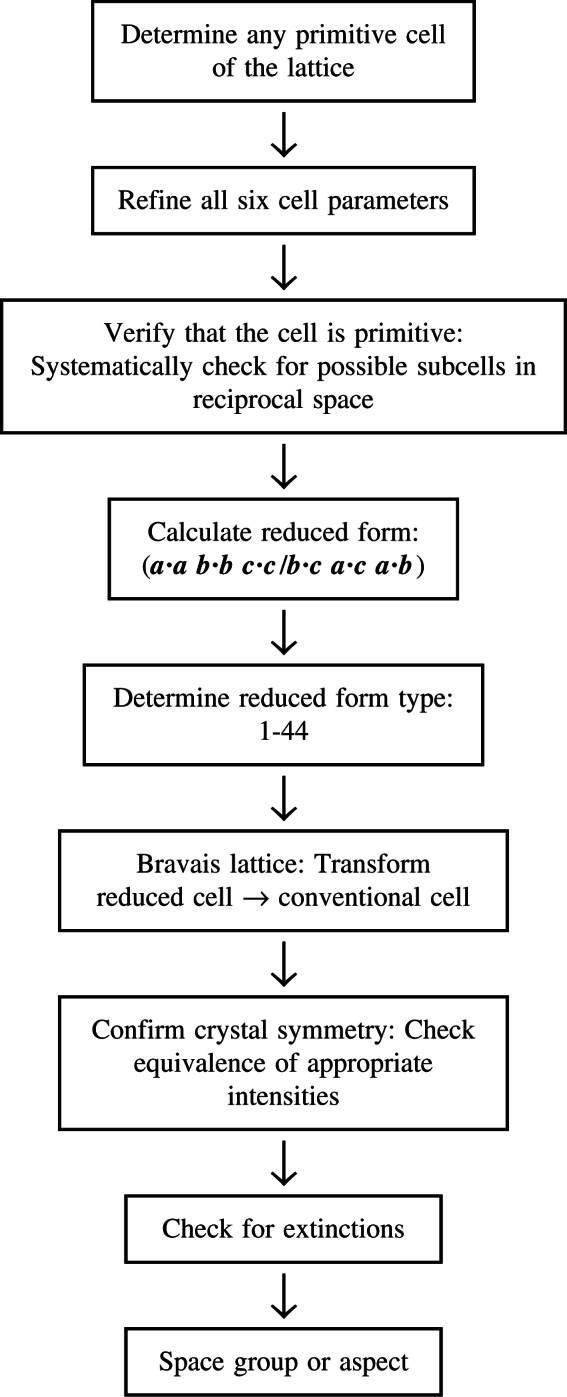
Symmetry determination: The reduced cell and reduced form as a routine tool.

The reduced cell is a standard cell that can be calculated [10] from any experimentally determined cell that defines the lattice. From this *unique* cell, one calculates the reduced form, which is then used to establish—by matching against the 44 reduced forms in Table 2—the metric lattice symmetry. Research with the crystallographic databases has proved that the metric and crystal symmetry are almost always the same. Furthermore, crystal symmetry can never exceed the lattice metric symmetry (e.g., if the metric symmetry is triclinic, the crystal symmetry must be triclinic). Consequently, as an integral part of the strategy for symmetry determination [19] outlined in Fig. 1, the experimentalist first establishes the metric symmetry and then the crystal symmetry. Thus in modern diffractometry, automated procedures use reduction procedures: (i) to establish if the compound has previously been investigated [8] and (ii) to obtain the metric symmetry.

### 4.2 Data Evaluation on Individual Entries

Because of the link between metric and crystal symmetry, the relationships in Table 2 are routinely used by the Crystallographic Data Centers in the critical evaluation of data. It is not uncommon for a compound to be reported in a space group of too low symmetry. A remarkable case of what can happen is illustrated in Table 6, in which, five independent determinations of 1,8-ter-pin are given [20–24] in chronological order (left to right). The first two papers report lattice parameters only, whereas the latter three describe full structure refinements. Reduction techniques prove that all five papers report the same compound (i.e., the reduced cells/compositions are identical). Note that for Lattice IV, the compound is described in a C-centered mono-clinic space group. However, inspection of Table 2, reveals that the reduced form (reduced form number = 16) corresponds to an F-centered orthorhombic lattice. In the final study (Lattice V), Marsh and Herbstein correct determination 4 and refine the compound in the F-centered orthorhombic lattice. It is instructive to note that the authors of determinations 4 and 5 make no reference to determination 3, which was originally correct!

### 4.3 Data Evaluation on Sets of Compounds

Experience in data evaluation has shown that experimentalists sometime miss the symmetry for centered Bravais lattices. In such cases, the compound is often reported in a crystal system of too low symmetry. For example, sometimes a crystalline compound that is rhombohedral is incorrectly reported in a C-centered monoclinic space group. Likewise an F-centered orthorhombic compound (e.g. determination 4 in Table 6) is sometimes incorrectly reported in a C-centered monoclinic space group. Using a crystallographic database one can systematically evaluate any given reduced from type. To evaluate this problem, all of the reduced forms in NIST Crystal Data [17] that correspond to orthorhombic centered lattices—i.e., reduced forms 8, 13, 16, 19, 23, 26, 36, 38, 40, 42—have been analyzed.

The results of the analysis are summarized in Table 7. The total number of compounds crystallizing in a given reduced form is given in the column labeled *ALL*. Those reported in the orthorhombic and monoclinic systems are presented in the columns labeled *orthorhombic* and *monoclinic*, respectively. As noted above the crystal and metric symmetry are highly correlated. Consequently, in Table 7, the compounds reported in the monoclinic system represent cases with potential error. Indeed a further analysis of selected cases from this category has revealed that many of these *monoclinic* compounds should have been reported in the orthorhombic system. For example, the selected compounds that were reported in monoclinic space groups but with metric orthorhombic F-centered lattices (reduced form 16, and 26) were shown using MISSYM [25,26] to have the higher crystal symmetry.

## 5. Derivative Lattices, Specialized Reduced Forms, and Lattice Metric Singularities

### 5.1 Derivative Lattices

Derivative lattice theory can be applied to the systematic study of lattices and to identification procedures. To understand and evaluate lattice symmetry, it is necessary to calculate and analyze the symmetry of the sets of associated derivative lattices. Definitions and treatment of derivative lattices are given in [27]. A convenient method for calculating the derivative sub- and superlattices of an *original* lattice of any desired multiplicity is outlined in reference [28] in an Appendix. (Multiplicity is defined as equal to the value of the determinant of the transformation matrix. Thus the value of the determinant times the volume of the *original* lattice is equal to the volume of the derivative lattice.) This method generates *unique* sets of upper triangular matrices for any given value of the determinant of the matrix. The required calculation can conveniently be done by the computer program NIST*LATTICE [10]. Table 8 gives the upper triangular matrices required to calculate the *unique* superlattices of multiplicities two, three, and four associated with an *original* lattice.

### 5.2 Specialized Reduced Forms

Sometimes a reduced form will exhibit specialization beyond that required for one of the 44 reduced forms in Table 2. Specialization can occur in two ways—a legitimate function of the crystal lattice or from an experimental error. For example, it can occur when one is dealing with an *original* lattice which is also a derivative lattice of a lattice with higher metric symmetry (see Tables 9 and 10). To recognize and characterize such specialization is desirable because many properties of crystals are not only related to the symmetry of the *original* lattice, but also to the symmetry of the associated derivative lattices.

#### 5.2.1 Specialized Reduced Forms Derived From an *Original* Cubic F Lattice

Specialized reduced forms can be generated by calculating derivative sublattices of an *original* cubic F lattice. The matrices (***X***) for calculating the sublattices are derived as noted from the ***Q*** matrices in Table 8. Table 9 shows sets of sublattices of an *original* cubic F lattice. Relations of the first seven sublattices in the Table to the *original* lattice are specified by a set of seven *unique* transformation matrices (***X***, |***X***| = 1/2), the next 13 are specified by 13 *unique* transformation matrices (***X***, |***X***| = 1/3), etc. The sublattices have different orientations with respect to the *original* lattices. In the general triclinic system, they also have seven different reduced forms. But as Table 9 illustrates, for the cubic F *original* cell, six of the sublattices (|***X***| = 1/2) have identical reduced forms (i.e., reduced form 23 = C-centered Bravais Lattice). This reduced form exhibits specialization as the relation (***b***·***b*** = 3 ***a***·***a***) is not required. In fact, as the table shows, all the sublattices with symmetry less than cubic have extra specialization in the reduced form.

#### 5.2.2 Specialized Reduced Forms Derived From an *Original* Cubic P Lattice

A second example of specialization can be generated by calculating derivative superlattices of an *original* cubic P lattice. The matrices (***Q***) for calculating the super-lattices are given in Table 8. Table 10 shows sets of superlattices of an *original* cubic P lattice. Relations of the first seven superlattices in the Table to the *original* lattice are specified by a set of seven *unique* transformation matrices (***Q***, |***Q***| = 2), the next 13 are specified by 13 *unique* transformation matrices (***Q***, |***Q***| = 3), etc. As the table shows, all the superlattices with symmetry less than cubic have extra specialization in the reduced form. In Tables 9 and 10, the reduced forms are represented in a normalized form—i.e., all the dot products are divided by smallest—so that extra specialization can readily be recognized.

### 5.3 Experimental Error Resulting From Omitted Nodes

Specialization sometimes occurs—especially if the *original* cell is of high symmetry—simply because the experimenter has determined a derivative rather than the *original* cell defining the lattice. Suppose a supercell of two times the volume of a primitive reciprocal cell has been selected. Depending on which nodes in the reciprocal lattice are omitted, one can obtain seven different superlattices of twice the volume of the *original* cell (note that some of the seven may be metrically identical—see Table 10—but have different orientations relative to the *original* lattice). Nevertheless, if a cell from a given superlattice is used as a basis cell, it is possible to calculate the set of seven sublattices of this superlattice. One of these is the true lattice.

### 5.4 Lattice Metric Singularities (LMS) in Powder Indexing

A lattice metric singularity (LMS) occurs when unit cells defining two or more lattices yield the identical set of *unique* calculated *d*-spacings [29]. In Table 11, a quaternary LMS is illustrated. In this highly unusual singularity, all four lattices are different Bravais lattices, each of which is characterized by a different reduced form. Furthermore, Lattices II–IV are all derivative sub-lattices of a cubic I-centered Bravais lattice and are all characterized by specialized reduced forms. Recently a ternary LMS was analyzed in which two of the lattices were hexagonal and one was orthorhombic. In this case, the two hexagonal lattices had the same volume and all three reduced forms were specialized. The existence of such singularities provides a warning to researchers who index powder patterns and rely on “Figures of Merit” as a sign of correctness.

## 6. Conclusion

Symmetry determination and identification procedures based on reduction have proved invaluable in crystallography and in the materials sciences. The symmetry determination strategies outlined herein are based on the fact that the reduced cell represents a *unique* standard cell that can be calculated from any cell of the lattice. This cell can be rigorously defined mathematically. Consequently, procedures based on reduction are highly reliable and are widely used in the scientific community—by individual scientists as well as by the crystallographic data centers. Because of their precise mathematical nature, they have been adapted to automated diffractometry and are routinely used as an integral part of structure-determination methodology worldwide.

Due somewhat to serendipity, however, the most significant and lasting value of this work is probably not reduction itself. Rather, reduction has played a key transition role in helping to move the discipline of crystallography in new directions with new insights. The research on reduction proved that there are excellent reasons for looking at the crystal lattice from an entirely different point of view. Consequently, with time, many other lattice-related papers followed, including papers on sublattices and superlattices, composite lattices, coincidence site lattices, and lattice-metric singularities in the indexing of powder patterns. At NIST, the mathematical analysis of lattices was pursued further and evolved to a matrix approach that offered a more abstract and powerful way to look at lattices and their properties. The matrix approach, in particular, has many applications including, for example, symmetry determination [3,30].

## Figures and Tables

**Table 1:** Conditions for a reduced cell^a^

The cell is specified by three noncoplanar vectors: ***a***, ***b***, ***c***. The cell matrix (***a·a b·b·c·c***/***b·c a·c a·b***) is defined by the dot products between these vectors.
A. Positive Reduced form, Type I cell, all angles < 90°
Main conditions:
***a***·***a*≤*b***·***b*≤*c***·***c***; ***b***·***c*≤**½***b***·***b***; ***a***·***c*≤**½***a***·***a***; ***a***·***b*≤** ½ ***a·a***
Special conditions:
(a) if ***a***·***a*** = ***b***·***b*** then ***b***·***c*≤*a***·***c***
(b) if ***b***·***b*** = ***c***·***c*** then ***a***·***c*≤*a***·***b***
(c) if ***b***·***c*** = ½ ***b***·***b*** then ***a***·***b* ≤** 2 ***a***·***c***
(d) if ***a***·***c*** = ½ ***a***·***a*** then ***a***·***b* ≤** 2 ***b***·***c***
(e) if ***a***·***b*** = ½ ***a***·***a*** then ***a***·***c* ≤** 2 ***b***·***c***
B. Negative reduced form, Type II cell, all angles ≥90°
Main conditions:
(a) ***a***·***a*≤*b***·***b*≤*c***·***c***; |***b***·***c***| **≤**½***b***·***b***; |***a***·***c***| **≤**½***a***·***a***; |***a***·***b***|**≤**½ ***a***·***a***
(b) (|***b***·***c***| + |***a***·***c***| + |***a***·***b***|) **≤** ½ (***a***·***a*** + ***b***·***b***)
Special conditions:
(a) if ***a***·***a*** = ***b***·***b*** then |***b***·***c***| **≤** |***a***·***c***|
(b) if ***b***·***b*** = ***c***·***c*** then |***a***·***c***| **≤** |***a***·***b***|
(c) if |***b***·***c***| = ½ ***b***·***b*** then ***a***·***b*** = 0
(d) if |***a***·***c***| = ½ ***a***·***a*** then ***a***·***b*** = 0
(e) if |***a***·***b***| = ½ ***a***·***a*** then ***a***·***c*** = 0
(f) if (|***b***·***c***| + |***a***·***c***| + |***a***·***b***|) = ½ (***a***·***a*** + ***b***·***b***) then ***a***·***a* ≤** 2 |***a***·***c***| + |***a***·***b***|

a To be reduced the cell must be in normal representation (type I or II) and all the main and special conditions for the given cell type must be satisfied. The main conditions are used to establish that a cell is based on the three shortest lattice translations. The special conditions are used to select a *unique* cell when two or more cells in the lattice have the same numerical values for the cell edges.

**Table 2:** Metric classification of the 44 reduced forms^a^. From the nature of the reduced form, one can determine the reduced form number, Bravais lattice, and the transformation matrix to the conventional cell

Reduced form No.	Reduced form matrix				Reduced form type	Bravais lattice		Cell transformation reduced → conventional
	First row	Second row						
	*a·a b·b c·c*	*b·c*	*a·c*	*a·b*				
*a*=*b*=*c*								
1	***a·a a·a a·a***	$\frac{a\cdot a}{2}$	$\frac{a\cdot a}{2}$	$\frac{a\cdot a}{2}$	+	Cubic	F	1 $\bar{1}$1/11 $\bar{1}$/ $\bar{1}$11
2	***a·a a·a a·a***	***b·c***	***b·c***	***b·c***	+	Rhombohedral	hR	1 $\bar{1}$0/ $\bar{1}$01/ $\bar{1}$ $\bar{1}$ $\bar{1}$
3	***a·a a·a a·a***	0	0	0	−	Cubic	P	100/010/001
4	***a·a a·a a·a***	−|b⋅c|	−|b⋅c|	−|b⋅c|	−	Rhombohedral	hR	1 $\bar{1}$0/ $\bar{1}$01/ $\bar{1}$ $\bar{1}$ $\bar{1}$
5	***a·a a·a a·a***	$-\frac{a\cdot a}{3}$	$-\frac{a\cdot a}{3}$	$-\frac{a\cdot a}{3}$	−	Cubic	I	101/110/011
6	***a·a a·a a·a***	$\frac{-a\cdot a+\left|a\cdot b\right|}{2}$	$\frac{-a\cdot a+\left|a\cdot b\right|}{2}$	−|a⋅b|	−	Tetragonal	I	011/101/110
7	***a·a a·a a·a***	−|b⋅c|	$\frac{-a\cdot a+\left|b\cdot c\right|}{2}$	$\frac{-a\cdot a+\left|b\cdot c\right|}{2}$	−	Tetragonal	I	101/110/011
8	***a·a a·a a·a***	−|b⋅c|	−|b⋅c|	−(|a⋅a|−|b⋅c|−|a⋅c|)	−	Orthorhombic	I	$\bar{1}$ $\bar{1}$0/ $\bar{1}$0 $\bar{1}$/0 $\bar{1}$ $\bar{1}$
*a*=*b*								
9	***a·a a·a c·c***	$\frac{a\cdot a}{2}$	$\frac{a\cdot a}{2}$	$\frac{a\cdot a}{2}$	+	Rhombohedral	hR	100/ $\bar{1}$10/ $\bar{1}$ $\bar{1}$3
10	***a·a a·a c·c***	***b·c***	***b·c***	***a·b***	+	Monoclinic	C^d^	110/1 $\bar{1}$0/00 $\bar{1}$
11	***a·a a·a c·c***	0	0	0	−	Tetragonal	P	100/010/001
12	***a·a a·a c·c***	0	0	$-\frac{a\cdot a}{2}$	−	Hexagonal	P	100/010/001
13	***a·a a·a c·c***	0	0	−|a⋅b|	−	Orthorhombic	C	110/ $\bar{1}$10/001
14	***a·a a·a c·c***	−|b⋅c|	−|b⋅c|	−|a⋅b|	−	Monoclinic	C^d^	110/ $\bar{1}$10/001
15	***a·a a·a c·c***	$-\frac{a\cdot a}{2}$	$-\frac{a\cdot a}{2}$	0	−	Tetragonal	I	100/010/112
16	***a·a a·a c·c***	−|b⋅c|	−|b⋅c|	−(a⋅a−2|b⋅c|)	−	Orthorhombic	F	$\bar{1}$ $\bar{1}$0/1 $\bar{1}$0/112
17	***a·a a·a c·c***	−|b⋅c|	−|b⋅c|	−(a⋅a−|b⋅c|−|a⋅c|)	−	Monoclinic	I^e^	$\bar{1}$0 $\bar{1}$/ $\bar{1}$ $\bar{1}$0/011
*b*=*c*								
18	***a·a b·b b·b***	$\frac{a\cdot a}{4}$	$\frac{a\cdot a}{2}$	$\frac{a\cdot a}{2}$	+	Tetragonal	I	0 $\bar{1}$1/1 $\bar{1}$ $\bar{1}$/100
19	***a·a b·b b·b***	***b·c***	$\frac{a\cdot a}{2}$	$\frac{a\cdot a}{2}$	+	Orthorhombic	I	$\bar{1}$00/0 $\bar{1}$1/ $\bar{1}$11
20	***a·a b·b b·b***	***b·c***	***a·c***	***a·c***	+	Monoclinic	C^b^	011/01 $\bar{1}$/ $\bar{1}$00
21	***a·a b·b b·b***	0	0	0	−	Tetragonal	P	010/001/100
22	***a·a b·b b·b***	$-\frac{b\cdot b}{2}$	0	0	−	Hexagonal	P	010/001/100
23	***a·a b·b b·b***	−|b⋅c|	0	0	−	Orthorhombic	C	011/0 $\bar{1}$1/100
24	***a·a b·b b·b***	$-\frac{b\cdot b-\frac{a\cdot a}{3}}{2}$	$-\frac{a\cdot a}{3}$	$-\frac{a\cdot a}{3}$	−	Rhombohedral	hR	121/0 $\bar{1}$1/100
25	***a·a b·b b·b***	−|b⋅c|	−|a⋅c|	−|a⋅c|	−	Monoclinic	C^b^	011/0 $\bar{1}$1/100
*a* **≤** *b* **≤** *c*								
26^g^	***a·a b·b c·c***	$\frac{a\cdot a}{4}$	$\frac{a\cdot a}{2}$	$\frac{a\cdot a}{2}$	+	Orthorhombic	F	100/ $\bar{1}$20/ $\bar{1}$02
27	***a·a b·b c·c***	***b·c***	$\frac{a\cdot a}{2}$	$\frac{a\cdot a}{2}$	+	Monoclinic	I^f^	0 $\bar{1}$1/ $\bar{1}$00/1 $\bar{1}$ $\bar{1}$
28	***a·a b·b c·c***	$\frac{a\cdot b}{2}$	$\frac{a\cdot a}{2}$	***a·b***	+	Monoclinic	C	$\bar{1}$00/ $\bar{1}$02/010
29	***a·a b·b c·c***	$\frac{a\cdot c}{2}$	***a·c***	$\frac{a\cdot a}{2}$	+	Monoclinic	C	100/1 $\bar{2}$0/00 $\bar{1}$
30	***a·a b·b c·c***	$\frac{b\cdot b}{2}$	$\frac{a\cdot b}{2}$	***a·b***	+	Monoclinic	C	010/01 $\bar{2}$/ $\bar{1}$00
31	***a·a b·b c·c***	***b·c***	***a·c***	***a·b***	+	Triclinic	P	100/010/001
32	***a·a b·b c·c***	0	0	0	−	Orthorhombic	P	100/010/001
33	***a·a b·b c·c***	0	−|a⋅c|	0	−	Monoclinic	P	100/010/001
34	***a·a b·b c·c***	0	0	−|a⋅b|	−	Monoclinic	P	$\bar{1}$00/00 $\bar{1}$/0 $\bar{1}$0
35	***a·a b·b c·c***	−|b⋅c|	0	0	−	Monoclinic	P	0 $\bar{1}$0/ $\bar{1}$00/00 $\bar{1}$
36	***a·a b·b c·c***	0	$-\frac{a\cdot a}{2}$	0	−	Orthorhombic	C	100/ $\bar{1}$0 $\bar{2}$/010
37	***a·a b·b c·c***	−|b⋅c|	$-\frac{a\cdot a}{2}$	0	−	Monoclinic	C^c^	102/100/010
38	***a·a b·b c·c***	0	0	$-\frac{a\cdot a}{2}$	−	Orthorhombic	C	$\bar{1}$00/120/00 $\bar{1}$
39	***a·a b·b c·c***	−|b⋅c|	0	$-\frac{a\cdot a}{2}$	−	Monoclinic	C^d^	$\bar{1}$ $\bar{2}$0/ $\bar{1}$00/00 $\bar{1}$
40	***a·a b·b c·c***	$-\frac{b\cdot b}{2}$	0	0	−	Orthorhombic	C	0 $\bar{1}$0/012/ $\bar{1}$00
41	***a·a b·b c·c***	$-\frac{b\cdot b}{2}$	−|a⋅c|	0	−	Monoclinic	C^b^	0 $\bar{1}$ $\bar{2}$/0 $\bar{1}$0/ $\bar{1}$00
42	***a·a b·b c·c***	$-\frac{b\cdot b}{2}$	$-\frac{a\cdot a}{2}$	0	−	Orthorhombic	I	$\bar{1}$00/0 $\bar{1}$0/112
43	***a·a b·b c·c***	$-\frac{b\cdot b-\left|a\cdot b\right|}{2}$	$-\frac{a\cdot a-\left|a\cdot b\right|}{2}$	−|a⋅b|	−	Monoclinic	I	$\bar{1}$00/ $\bar{1}$ $\bar{1}$ $\bar{2}$/0 $\bar{1}$0
44	***a·a b·b c·c***	−|b⋅c|	−|a⋅c|	−|a⋅b|	−	Triclinic	P	100/010/001

a Based on Table 5.1.3.1 of the International Tables for X-Ray Crystallography [9] and published revisions [11,12,13].

$\begin{array}{c} {\;}^{b}\text{If}\;a\cdot a<\;4\left|a\cdot c\right|\\ {\;}^{c}\text{If}\;b\cdot b<\;4\left|b\cdot c\right|\\ {\;}^{d}\text{If}\;c\cdot c<\;4\left|b\cdot c\right| \end{array}\}\;\begin{array}{l} \;\text{Premultiply table matrix by}\;00\bar{1}/010/101\;\left(\text{I centered}\right). \end{array}$.

$\begin{array}{c} {\;}^{e}\text{If}\;3a\cdot a<c\cdot c+\;2\left|a\cdot c\right|\\ {\;}^{f}\text{If}\;3b\cdot b<c\cdot c+\;2\left|b\cdot c\right| \end{array}\}\begin{array}{l} \text{Premultiply table matrix by}\;\bar{1}0\bar{1}/010/100\;\left(\text{C centered}\right). \end{array}$.

g No required relationships between symmetrical scalars for reduced forms 26–44.

**Table 3:** Reduced form frequency for 133 613 organic compounds

Reducedform No.^a^	Bravais lattice		Count	% Total
1	Cubic	F	165	0.12
2	Rhombohedral	R	324	0.24
3	Cubic P	P	544	0.41
4	Rhombohedral	R	441	0.33
5	Cubic	I	137	0.10
6	Tetragonal	I	123	0.09
7	Tetragonal	I	231	0.17
8	Orthorhombic	I	28	0.02
9	Rhombohedral	R	281	0.21
10	Monoclinic	C/I	2151	1.61
11	Tetragonal	P	1499	1.12
12	Hexagonal	P	921	0.69
13	Orthorhombic	C	737	0.55
14	Monoclinic	C/I	1277	0.96
15	Tetragonal	I	304	0.23
16	Orthorhombic	F	265	0.20
17	Monoclinic	I/C	765	0.57
18	Tetragonal	I	504	0.38
19	Orthorhombic	I	188	0.14
20	Monoclinic	C/I	667	0.50
21	Tetragonal	P	1154	0.86
22	Hexagonal	P	801	0.60
23	Orthorhombic	C	327	0.24
24	Rhombohedral	R	351	0.26
25	Monoclinic	C/I	398	0.30
26	Orthorhombic	F	386	0.29
27	Monoclinic	I/C	2350	1.76
28	Monoclinic	C	110	0.08
29	Monoclinic	C	436	0.33
30	Monoclinic	C	141	0.11
31	Triclinic	P	13959	10.45
32	Orthorhombic	P	27154	20.32
33	Monoclinic	P	15937	11.93
34	Monoclinic	P	20554	15.38
35	Monoclinic	P	20048	15.00
36	Orthorhombic	C	237	0.18
37	Monoclinic	C/I	1201	0.90
38	Orthorhombic	C	442	0.33
39	Monoclinic	C/I	2718	2.03
40	Orthorhombic	C	232	0.17
41	Monoclinic	C/I	393	0.29
42	Orthorhombic	I	136	0.10
43	Monoclinic	I	138	0.10
44	Triclinic	P	12458	9.32

a Reduced form number (see Table 2).

**Table 4:** Population frequency for the 14 Bravais lattices for 133 613 organic compounds

	Bravais lattice		Count	% Total
1	Triclinic	P	26417	19.77
2	Monoclinic	P	56539	42.32
3	Monoclinic	C/I	12745	9.54
4	Orthorhombic	P	27154	20.32
5	Orthorhombic	C	1975	1.48
6	Orthorhombic	I	352	0.26
7	Orthorhombic	F	651	0.49
8	Rhombohedral	P	1397	1.05
9	Tetragonal	P	2653	1.99
10	Tetragonal	I	1162	0.87
11	Hexagonal	P	1722	1.29
12	Cubic	P	544	0.41
13	Cubic	I	137	0.10
14	Cubic	F	165	0.12

**Table 5:** Population frequency by crystal system for 133 613 organic compounds

	Bravais lattice	Count	% Total
1	Triclinic	26417	19.77
2	Monoclinic	69284	51.85
3	Orthorhombic	30132	22.55
4	Rhombohedral	1397	1.05
5	Tetragonal	3815	2.86
6	Hexagonal	1722	1.29
7	Cubic	846	0.63

**Table 6:** Crystallographic parameters reported for 1,8-terpin (C_10_H_20_O_2_·H_2_O) in Refs. [20–24]. Lattice IV was incorrectly reported as monoclinic. However, the reduced form (No. 16) for Cell 4 shows that the lattice is metrically F-centered orthorhombic. Numbers in parentheses represent standard deviations

No.	1[20]	2[21]	3[22]	4[23]	5[24]

	Lattice I	Lattice II	Lattice III	Lattice IV	Lattice V
	Orthorhombic F	Orthorhombic F	Orthorhombic F	Monoclinic C	Orthorhombic F

Literature cells					

Cell	Cell 1	Cell 2	Cell 3	Cell 4	Cell 5
*a*(Å)	18.51	18.60	10.930(2)	10.912(3)	18.421
*b*(Å)	22.87	23.00	18.425(5)	22.791(4)	22.791
*c*(Å)	10.96	10.86	22.791(6)	10.705(2)	10.912
*α* (°)	90.0	90.0	90.0	90.0	90.0
*β* (°)	90.0	90.0	90.0	120.64	90.0
*γ* (°)	90.0	90.0	90.0	90.0	90.0
*V*(Å^3^)	4639.6	4645.9	4589.8	2290.6	4581.2
Sp. Gr.	F*	Fdd2	Fdd2	Cc	Fdd2
Yr. Pub.	1951	1965	1982	1986	1988

Reduced cells					

Cell	R1	R2	R3	R4	R5
*a*(Å)	10.76	10.769	10.712	10.705	10.705
*b*(Å)	10.76	10.769	10.712	10.705	10.705
*c*(Å)	12.68	12.718	12.638	12.634	12.634
*α* (°)	102.72	102.43	102.74	102.71	102.71
*β* (°)	102.72	102.43	102.74	102.71	102.71
*γ* (°)	118.74	119.44	118.65	118.72	118.72
*V*(Å^3^)	1159.9	1161.5	1147.4	1145.3	1145.3

Reduced forms					

Form	F1	F2	F3	F4	F5
***a***·***a***	115.68	115.98	114.74	114.60	114.60
***b***·***b***	115.68	115.98	114.74	114.60	114.60
***c***·***c***	160.79	161.74	159.72	159.62	159.62
***b***·***c***	−30.03	−29.48	−29.87	−29.77	−29.77
***a***·***c***	−30.03	−29.48	−29.87	−29.77	−29.77
***a***·***b***	−55.62	−57.00	−55.00	−55.06	−55.06
Form No.	16	16	16	16	16

**Table 7:** Analysis of the centered orthorhombic Bravais lattices in NIST Crystal Data. The total number of organic compounds in all 44 reduced forms is 133 613 out of which 2978 have centered orthorhombic lattices

No.	Bravais lattice	Reduced form No.	ALL^a^	Monoclinic^b^	Orthorhombic^c^	% Lower symmetry
1	OI^d^	8	28	3	25	10.7
2	OC	13	737	159	578	21.6
3	OF	16	265	22	243	8.3
4	OI	19	188	19	169	10.1
5	OC	23	327	158	169	48.3
6	OF	26	386	22	364	5.7
7	OC	36	237	109	128	46.0
8	OC	38	442	154	288	34.8
9	OC	40	232	139	93	59.9
10	OI	42	136	11	125	8.1

		Sum =	2978	796	2182	

a Total number of compounds with specified reduced form.

b Number of compounds reported as monoclinic. For these compounds, the crystal symmetry is less than the metric symmetry.

c Number of compounds reported as orthorhombic. For these compounds, the crystal symmetry is equal to the metric symmetry.

d Orthorhombic I- centered (i.e., 1st letter = system; 2nd letter = centering).

**Table 8:** *Unique* ***Q*** matrices [28,10] generating 7, 13, and 35 super-lattices for |***Q***| = 2, 3, and 4, respectively. The unique matrices generating 7, 13, 35 sublattices for |***X***| = ½, ⅓, ¼ are obtained by taking the transpose of the inverse of the matrices given for the superlattices. For each value of |***Q***| or |***X***|, the matrices can be applied to any primitive cell of the *original* lattice, but they must be applied to the same cell

|Q|=2	100 / 010 / 002	100 / 011 / 002	101 / 010 / 002
	101 / 011 / 002	100 / 020 / 001	110 / 020 / 001
	200 / 010 / 001		
|Q|=3	100 / 010 / 003	100 / 011 / 003	100 / 012 / 003
	101 / 010 / 003	101 / 011 / 003	101 / 012 / 003
	102 / 010 / 003	102 / 011 / 003	102 / 012 / 003
	100 / 030 / 001	110 / 030 / 001	120 / 030 / 001
	300 / 010 / 001		
|Q|=4	100 / 010 / 004	100 / 011 / 004	100 / 012 / 004
	100 / 013 / 004	101 / 010 / 004	101 / 011 / 004
	101 / 012 / 004	101 / 013 / 004	102 / 010 / 004
	102 / 011 / 004	102 / 012 / 004	102 / 013 / 004
	103 / 010 / 004	103 / 011 / 004	103 / 012 / 004
	103 / 013 / 004	100 / 020 / 002	100 / 021 / 002
	101 / 020 / 002	101 / 021 / 002	110 / 020 / 002
	110 / 021 / 002	111 / 020 / 002	111 / 021 / 002
	100 / 040 / 001	110 / 040 / 001	120 / 040 / 001
	130 / 040 / 001	200 / 010 / 002	200 / 011 / 002
	201 / 010 / 002	201 / 011 / 002	200 / 020 / 001
	210 / 020 / 001	400 / 010 / 001	

**Table 9:** Specialized derivative sublattices (derived from a Cubic F lattice). All sublattices with symmetry less than cubic have extra specialization in their reduced form

Reduced form No.	Reduced form^a^						Bravais lattice		No. lattices	*V*/*V*_org_
	First row			Second row						
	*a·a*	*b·b*	*c·c*	*b·c*	*a·c*	*a·b*				
*Original* lattice										

1	2	2	2	1	1	1	Cubic	F	1	1

7 Sublattices |***X***| = 1/2										

3	1	1	1	0	0	0	Cubic	P	1	1/2
23	1	3	3	−1	0	0	Orthorhombic	C	6	1/2

13 Sublattices |***X***| = 1/3										

12	2	2	4	0	0	−1	Hexagonal	P	4	1/3
18	4	10	10	1	2	2	Tetragonal	I	3	1/3
19	2	14	14	5	1	1	Orthorhombic	I	6	1/3

35 Sublattices |***X***| = 1/4										

5	3	3	3	−1	−1	−1	Cubic	I	1	1/4
9	2	2	6	1	1	1	Rhombohedral	P	4	1/4
11	1	1	2	0	0	0	Tetragonal	P	3	1/4
21	1	4	4	0	0	0	Tetragonal	P	3	1/4
23	1	12	12	−4	0	0	Orthorhombic	C	6	1/4
26	4	5	9	1	2	2	Orthorhombic	F	6	1/4
33	3	4	11	0	−1	0	Monoclinic	P	12	1/4

a The reduced forms have been normalized.

**Table 10:** Specialized derivative superlattices (derived from a Cubic P *original* lattice). All superlattices with symmetry less than cubic have *extra* specialization in their reduced form

Reduced form No.	Reduced form^a^						Bravais lattice		No. lattices	*V*/*V*_org_
	First row			Second row						
	*a·a*	*b·b*	*c·c*	*b·c*	*a·c*	*a·b*				
*Original* lattice										

1	1	1	1	0	0	0	Cubic	P	1	1

7 Superlattices |***Q***| = 2										

1	2	2	2	1	1	1	Cubic	F	1	2
11	1	1	4	0	0	0	Tetragonal	P	3	2
21	1	2	2	0	0	0	Tetragonal	P	3	2

13 Superlattices |***Q***| = 3										

11	1	1	9	0	0	0	Tetragonal	P	3	3
12	2	2	3	0	0	−1	Hexagonal	P	4	3
40	1	2	5	−1	0	0	Orthorhombic	C	6	3

35 Superlattices |***Q***| = 4										

5	3	3	3	−1	−1	−1	Cubic	I	1	4
9	2	2	6	1	1	1	Rhombohedral	P	4	4
11	1	1	2	0	0	0	Tetragonal	P	3	4
11	1	1	16	0	0	0	Tetragonal	P	3	4
15	2	2	5	−1	−1	0	Tetragonal	I	3	4
21	1	4	4	0	0	0	Tetragonal	P	3	4
23	2	3	3	−1	0	0	Orthorhombic	C	6	4
32	1	2	8	0	0	0	Orthorhombic	P	6	4
40	1	4	5	**−**2	0	0	Orthorhombic	C	6	4

a The reduced forms have been normalized.

**Table 11:** Quaternary lattice metric singularity. The four lattices yield the same set of *unique* calculated *d*-spacings. For each lattice, the table gives the conventional cell along with the corresponding reduced cell and normalized reduced form. The normalized reduced forms reveal extra specialization in forms F2–F4

	Lattice I	Lattice II	Lattice III	Lattice IV
	Cubic I	Tetragonal P	Orthorhombic F	Orthorhombic P
Conventional cells				

Cell	Cell 1	Cell 2^a^	Cell 3^b^	Cell 4^c^
*a*(Å)	10.0000	7.0711	4.7140	3.5355
*b*(Å)	10.0000	7.0711	10.0000	5.0000
*c*(Å)	10.0000	5.0000	14.1421	7.0711
*α* (°)	90.0	90.0	90.0	90.0
*β* (°)	90.0	90.0	90.0	90.0
*γ* (°)	90.0	90.0	90.0	90.0
*V*(Å^3^)	1000.0	250.0	666.67	125.0

Reduced cells				

Cell	R1	R2^d^	R3^e^	R4^f^
*a*(Å)	8.6603	5.0000	4.7140	3.5355
*b*(Å)	8.6603	7.0711	5.5277	5.0000
*c*(Å)	8.6603	7.0711	7.4536	7.0711
*α* (°)	109.471	90.0	82.251	90.0
*β* (°)	109.471	90.0	71.565	90.0
*γ* (°)	109.471	90.0	64.761	90.0
*V*(Å^3^)	500.0	250.0	166.67	125.0

Normalized reduced forms				

Form	F1	F2	F3	F4
***a·a***	3	1	4	1
***b·b***	3	2	5.5	2
***c·c***	3	2	10	4
***b·c***	−1	0	1	0
***a·c***	−1	0	2	0
***a·b***	−1	0	2	0
Form No.	5	21	26	32

Transformations

a Cell 2 → Cell 1 [ 0 0 2 / 1− 1 0 / 1 1 0 ]∆ = 4.

b Cell 3 → Cell 1 [1/2−2/3−1/2 / 2 1/3 0 / 1/2 −2/3 1/2]∆ = 3/2.

c Cell 4 → Cell 1 [ 0 2 0 / 2 0 1 / 2 0− 1 ]∆ = 8.

d R2 → R1 [ 1 −1 0 / −1 0 1 / −1 0 −1 ]∆ = 2.

e R3 → R1 [ 1 1 0 / −2 1 0 / 0 −1 1 ]∆ = 3.

F R4 → R1 [ 0 −1 −1 / 2 1 0 / 0 −1 1 ]∆ = 4.
